# Headspace Volatile Organic Compound Profiling of Pleural Mesothelioma and Lung Cancer Cell Lines as Translational Bridge for Breath Research

**DOI:** 10.3389/fonc.2022.851785

**Published:** 2022-05-06

**Authors:** Eline Janssens, Zoë Mol, Lore Vandermeersch, Sabrina Lagniau, Karim Y. Vermaelen, Jan P. van Meerbeeck, Christophe Walgraeve, Elly Marcq, Kevin Lamote

**Affiliations:** ^1^Laboratory of Experimental Medicine and Pediatrics, University of Antwerp, Antwerp, Belgium; ^2^Infla-Med Center of Excellence, University of Antwerp, Antwerp, Belgium; ^3^Department of Green Chemistry and Technology, Environmental Organic Chemistry and Technology (EnVOC) Research Group, Ghent University, Ghent, Belgium; ^4^Department of Internal Medicine and Pediatrics, Ghent University, Ghent, Belgium; ^5^Department of Respiratory Medicine, Ghent University Hospital, Ghent, Belgium; ^6^Tumor Immunology Lab, Ghent University, Ghent, Belgium; ^7^Department of Pulmonology and Thoracic Oncology, Antwerp University Hospital, Edegem, Belgium; ^8^Center for Oncological Research (CORE), Integrated Personalized and Precision Oncology Network (IPPON), University of Antwerp, Antwerp, Belgium

**Keywords:** mesothelioma, lung cancer, biomarkers, volatile organic compounds, headspace analysis

## Abstract

**Introduction:**

Malignant pleural mesothelioma (MPM) is a lethal cancer for which early-stage diagnosis remains a major challenge. Volatile organic compounds (VOCs) in breath proved to be potential biomarkers for MPM diagnosis, but translational studies are needed to elucidate which VOCs originate from the tumor itself and thus are specifically related to MPM cell metabolism.

**Methods:**

An *in vitro* model was set-up to characterize the headspace VOC profiles of six MPM and two lung cancer cell lines using thermal desorption-gas chromatography-mass spectrometry. A comparative analysis was carried out to identify VOCs that could discriminate between MPM and lung cancer, as well as between the histological subtypes within MPM (epithelioid, sarcomatoid and biphasic).

**Results:**

VOC profiles were identified capable of distinguishing MPM (subtypes) and lung cancer cells with high accuracy. Alkanes, aldehydes, ketones and alcohols represented many of the discriminating VOCs. Discrepancies with clinical findings were observed, supporting the need for studies examining breath and tumor cells of the same patients and studying metabolization and kinetics of *in vitro* discovered VOCs in a clinical setting.

**Conclusion:**

While the relationship between *in vitro* and *in vivo* VOCs is yet to be established, both could complement each other in generating a clinically useful breath model for MPM.

## 1 Introduction

Malignant pleural mesothelioma (MPM) is a rare and lethal thoracic cancer, arising from the mesothelial cells lining the lungs and chest wall. A clear causal relationship has been established between asbestos exposure and MPM development (1). Although the use of asbestos was banned in most Western countries many years ago, the incidence of MPM is expected to increase during the next years in numerous countries due to the long latency period (of up to 50 years) between first exposure and the onset of symptoms (1, 2). Moreover, people who have been exposed to asbestos are also at higher risk of developing lung cancer, which even increases synergistically when combined with tobacco smoke exposure (3).

With a five-year survival rate of less than 5%, prognosis for MPM remains very poor (4). MPM is classified into three major histological subtypes (epithelioid, sarcomatoid and biphasic) with a non-epithelioid histology being an unfavorable prognostic factor (5). One of the major challenges concerning this type of cancer is its early-stage diagnosis. The delayed onset of (non-specific) symptoms causes MPM to be mainly diagnosed in an advanced stage, which limits curative treatment options. The diagnostic process can be complex, as radiological findings represent a wide range of manifestations and may mimic lung cancer, for example, requiring histopathological confirmation to reach a definite diagnosis (5, 6). With standard-of-care combination chemotherapy, median survival in selected patients is around 13 months, which can be modestly improved up to 18 months with either the addition of the anti-angiogenic agent bevacizumab or dual immunotherapy with ipilimumab and nivolumab (7). Diagnosing MPM in an earlier stage is hypothesized to improve patient survival (8). It is therefore important to develop reliable early diagnostic tools, which are currently lacking, that would allow screening and surveillance of individuals who have been exposed to asbestos (9). Although much effort has been put into finding suitable blood biomarkers such as mesothelin, high-mobility group box protein 1 and fibulin-3, this has not yet led to a clinically useful one (10).

The analysis of exhaled breath on the other hand, is an emerging research field in this quest for reliable, early-stage biomarkers. Several clinical studies have proven that volatile organic compounds (VOCs), present in breath, could adequately distinguish MPM patients from asbestos-exposed control groups, which was also demonstrated in our previous work (11–15). However, the clinical implementation of these VOC-based diagnostic models is hampered due to the lack of validation and biological translation studies. To gain knowledge about the biochemical origin and metabolization of these VOC biomarkers, it is crucial to investigate VOC production at the cellular level (16). By studying pure populations of tumor cells, the contribution of tumor-associated stromal cells (e.g. immune infiltrates, fibroblasts) or the microbiome (bacterial, fungal or others) to VOC profiles can be eliminated, making it easier to identify which VOCs are truly tumor cell-derived. Additionally, *in vitro* VOC research allows investigation of potential biomarkers while bypassing confounding factors that could influence (breath) VOC profiles in clinical settings (age, diet, medication use, smoking status etc.) (16). Hence, an *in vitro* approach makes it possible to pinpoint specific tumor cell-derived VOCs, which can improve the current discriminative models. Presently there are many of this type of *in vitro* studies for lung cancer (17), but for MPM these are sparse as only two studies reported data on *in vitro* headspace analysis of MPM cells (18, 19).

To learn more about the cellular origin of breath VOCs, the goal of this study was to analyze and characterize the VOC profiles in the headspace of six different MPM cell lines, representing the three major histological subtypes of MPM (epithelioid, sarcomatoid and biphasic), and two lung cancer cell lines, using thermal desorption-gas chromatography-mass spectrometry (TD-GC-MS). A comparative analysis was carried out to identify VOCs that could discriminate between MPM and lung cancer, as well as between the histological subtypes within MPM. This approach could discover compounds that arise from MPM cells and have the potential to be diagnostic or, in extension, even prognostic MPM biomarkers.

## 2 Materials and Methods

### 2.1 Cell Culture

Six different human MPM cell lines were used, representing the major histological subtypes of MPM: two sarcomatoid (NCI-H2731 and H-MESO-1), two epithelioid (NCI-H2795 and NCI-H2818) and two biphasic (NKI04 and MSTO-211H) (**Supplementary Figure S1A**). To assess the specificity of the VOCs, two non-small cell lung cancer (NSCLC) cell lines (NCI-H2228 and NCI-H1975), representing the most common type of lung cancer, were also included. The NCI-H2731, NCI-H2795, NCI-H2818 and NKI04 cell lines were kindly provided by Prof. Dr. Paul Baas from the Netherlands Cancer Institute (NKI, Amsterdam, The Netherlands). The MSTO-211H, NCI-H2228 and NCI-H1975 cell lines were purchased from ATCC (Manassas, Virginia, USA). The H-MESO-1 cell line was purchased from CLS Cell Lines Service GmbH (Eppelheim, Germany). All cell lines tested negative for mycoplasma contamination through routine testing.

All cell lines were cultivated under standard conditions at 37°C and 5% CO_2_. NCI-H2818, NCI-H2795, NCI-H2731 and NKI04 cells were grown in DMEM/F-12 Glutamax™ supplemented with 10% fetal bovine serum (FBS), penicillin (100 000 units/L) and streptomycin (100 mg/L). H-MESO-1, MSTO-211H, NCI-H2228 and NCI-H1975 cells were grown in RPMI 1640 supplemented with 10% FBS, penicillin (100 000 units/L), streptomycin (100 mg/L) and L-glutamine (2 mM). Upon reaching 70-90% confluence, the cells were harvested with 0,05% trypsin and seeded in new culture flasks to increase the number of cells.

Before sampling, the cells were seeded in 175 cm^2^ culture flasks and incubated for exactly 48 hours. The seeding ratio was determined from the growth rate of the different cell lines, so confluence would be reached after the 48-hour incubation period. Culture flasks with blank medium (complete DMEM/F-12 Glutamax™ or RPMI 1640), incubated under the same experimental conditions, were used as controls. At least five replicates of each histological subtype and control type were used.

### 2.2 Headspace Sampling

Sampling was performed in a laminar flow cabinet to minimize environmental contamination. Headspace VOCs were collected on Tenax^®^GR sorbent tubes (Markes, Llantrisant, UK), after the 48-hour incubation period, by drawing the headspace air through the sorbent tube at a flow rate of 100 ml/min for 16 min (**Supplementary Figure S1B**). After sampling, the tubes were immediately sealed with brass storage caps fitted with PTFE ferrules and stored in a glass container, protected from air and light. Prior to sampling, these sorbent tubes were conditioned for one hour at 300°C while being flushed with helium (50 ml/min) and loaded with 10.8 ng toluene-d8 as internal standard (20). Immediately after sampling, both cell number and viability in each culture flask were assessed using the trypan blue exclusion method (TC20™ automated cell counter, Bio-Rad).

### 2.3 VOC Analysis by TD-GC-MS

After sampling, headspace VOCs were desorbed from the Tenax^®^GR sorbent tubes using a Unity series 2 Thermal Desorption system (Markes, Llantrisant, UK). First, the sorbent tubes were dry purged for 4 min at 20 ml/min to remove any water and pre-purged with helium for 2 min at 20 ml/min to remove any air which could cause oxidation. Next, the VOCs were desorbed from the tubes by heating them to 260°C for 10 min under a helium flow of 20 ml/min. The analytes were then refocused on a cooled microtrap (-10°C) filled with 29 mg Tenax^®^TA 35/60 and 28.3 mg Carbograph 1TD 40/60 sorbent. The microtrap was desorbed by flash-heating at 280°C for 3 min. The analytes were then carried to the capillary GC column by a helium-flow, after splitting the flow at 10 ml/min (**Supplementary Figure S1C**). The flow path was heated to 130°C. The GC (Focus GC, Thermo Scientific, Milan, Italy) contains a 30 m FactorFour VF-1ms low bleed bounded phase capillary GC column (Varian, Sint-Katelijne-Waver, Belgium; 100% polydimethylsiloxane, internal diameter 0.25 mm, film thickness 1 µm). The temperature of this column was adjusted in four steps: initially, the temperature was set at 35°C during the first 10 min after injection. Next, the temperature started to increase with 2°C/min until a temperature of 60°C was reached. Subsequently, the temperature was increased to 170°C at 8°C/min and finally to 240°C at 15°C/min which was maintained for 10 min. The transfer line to the mass spectrometer was heated to 240°C. The DSQII Single Quadrupole mass spectrometer (Thermo Scientific, Austin, TX, USA) uses electron ionization (70 eV). Ions with a mass-to-charge (m/z) ratio from 29 to 300 were recorded in full scan mode (200 ms/scan).

### 2.4 Data Processing and Statistical Analysis

Chromatograms and mass spectra were processed using Thermo XCalibur 2.2 software. Compounds were tentatively identified based on their retention time, fragmentation patterns and spectral match with the National Institute of Standards and Technology (NIST) Mass Spectral (MS) search V2.0 database. The internal standard toluene-d8 was used to correct for variability in TD-GC-MS performance. Hence, the peak area relative to the internal standard (RPA) was determined for every compound and used for further processing. The quality of the dataset was examined by checking the reproducibility of the replicates. Compounds with a relative standard deviation exceeding 30% in ≥60% of the sample types were discarded (21). Internal standard-based normalization and scaling to unit variance were applied to the data prior to statistical analysis.

Statistical analysis was performed using R software with the R Studio interface. Before comparing the VOC profiles of the different cell lines, a background correction was applied to correct for the background signals originating from the two cell culture media (full DMEM/F-12 Glutamax™ and RPMI 1640 medium) and cell culture flasks used (22). This was done by subtracting the average RPA of each VOC of the corresponding media samples (control samples) from the RPA of each cell culture sample. Next, unsupervised methods were applied including principal component analysis (PCA) and hierarchical clustering analysis (HCA) to explore the data. Differences in VOC profiles between sample types were investigated using the supervised method least absolute shrinkage and selection operator (lasso) regression. Different lasso classification models were created: (1) MPM versus lung cancer (one model), (2) MPM histological subtypes versus lung cancer (three models) and (3) MPM histological subtype versus MPM histological subtype (three models). The *glmnet* R-package (v2.0-2) was used for fitting binominal lasso logistic models. The constructed discrimination models were validated by leave-one-out cross-validation. For visualization, receiver operating characteristic (ROC) curves were created followed by estimation of the model characteristics [sensitivity, specificity, accuracy and area under the curve (AUC_ROC_)] with their 95% confidence intervals. Furthermore, the number of times (folds) a VOC was selected by the lasso regressions was also determined. Variables were considered as important in the discrimination when selected in a large proportion of folds (>80%).

## 3 Results

### 3.1 Cell Viability

The average viability (%) and number of viable cells (± standard deviation) of the six MPM and two NSCLC cell lines are shown in **Table 1**. The average cell viability ranged from 87.2 ± 13.3% to 100 ± 0.0%, showing that cell culture conditions did not substantially affect the viability of the cells. The released VOCs thus mainly come from living cells, reflecting the normal metabolism of the analyzed cell lines.

**Table 1:** Average cell viability (%) and number of viable cells (x10^6^) of the replicates of the different cell lines after 48 hours of incubation [n=5, except for NKI04 (n=3), NCI-H2228 (n=3) and NCI-H1975 (n=2)].

Cell line	Cell type	Average viability (%)	Average number of viable cells (x10^6^)
**H-MESO-1**	Sarcomatoid MPM	98.4 ± 0.5	21.3 ± 3.4
**NCI-H2731**	Sarcomatoid MPM	98.0 ± 2.9	6.8 ± 2.0
**NCI-H2795**	Epithelioid MPM	98.8 ± 0.8	6.1 ± 1.3
**NCI-H2818**	Epithelioid MPM	87.2 ± 13.3	7.6 ± 0.7
**MSTO-211H**	Biphasic MPM	99.0 ± 1.0	5.8 ± 1.9
**NKI04**	Biphasic MPM	99.3 ± 1.2	3.0 ± 0.5
**NCI-H2228**	NSCLC	100 ± 0.0	9.8 ± 1.7
**NCI-H1975**	NSCLC	99.0 ± 0.0	6.0 ± 0.6

MPM, malignant pleural mesothelioma; NSCLC, non-small cell lung cancer.

Average values are presented with their standard deviation.

### 3.2 Headspace VOC Profiling

#### 3.2.1 Data Exploration: PCA and HCA

In total, 277 VOC peaks were selected in the obtained chromatograms of which 77 could be identified. These 77 identified compounds could be assigned to eleven different chemical classes: alcohols, aldehydes, aliphatic hydrocarbons, aromatic hydrocarbons, esters, halogenated compounds, ketones, nitrogen compounds, siloxanes, sulphides and terpenes (**Supplementary Table S1**). The unidentified compounds were named according to their retention time (e.g. RT_17.65). For thirteen of the 277 VOCs, the relative standard deviation of their RPA exceeded 30% in ≥60% of the sample types, indicating low stability over the replicates. Therefore, they were disregarded in further analysis.

**Figure 1:**
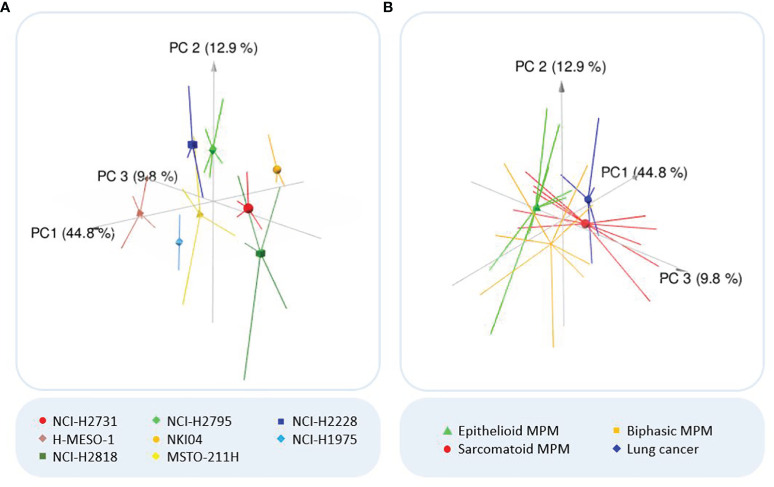
Outcome principal component analysis (PCA). **(A)** Three-dimensional PCA plot of all analyzed cell lines. Colors of the cell lines: light/dark green = epithelioid MPM; red/brown = sarcomatoid MPM; yellow/orange = biphasic MPM; dark/light blue = lung cancer. **(B)** Three-dimensional PCA plot of the MPM subhistologies and lung cancer. PCA reduces the large number of variables to a few principal components (PCs), which account for the most variation in the data. As the first three PCs account for most of the variation, the three-dimensional PCA plot shows clusters of samples based on their similarity. Consequently, the more distant the samples are, the more they differ (according to the variation explained by the axes). The symbols indicate the centroids of the sample replicates.

After pre-processing and background correction, unsupervised data exploration was performed by PCA and HCA to visualize the differences between the VOCs present in the headspace of the various cell lines. The largest variation in the samples is explained by PC1 (44.8%), with PC2 and PC3 explaining an additional 12.9% and 9.8% of the total variation in the data respectively (**Figure 1**). Although some overlap is seen between a few individual cell lines, indicating partial similarity between the VOC profiles, some separation could still be observed, meaning there are also differences in VOCs (**Figure 1A**). When the cell lines of the same histology are pooled, the groups are closer together and more overlap can be observed, but the separation is still noticeable (**Figure 1B**).

**Figure 2:**
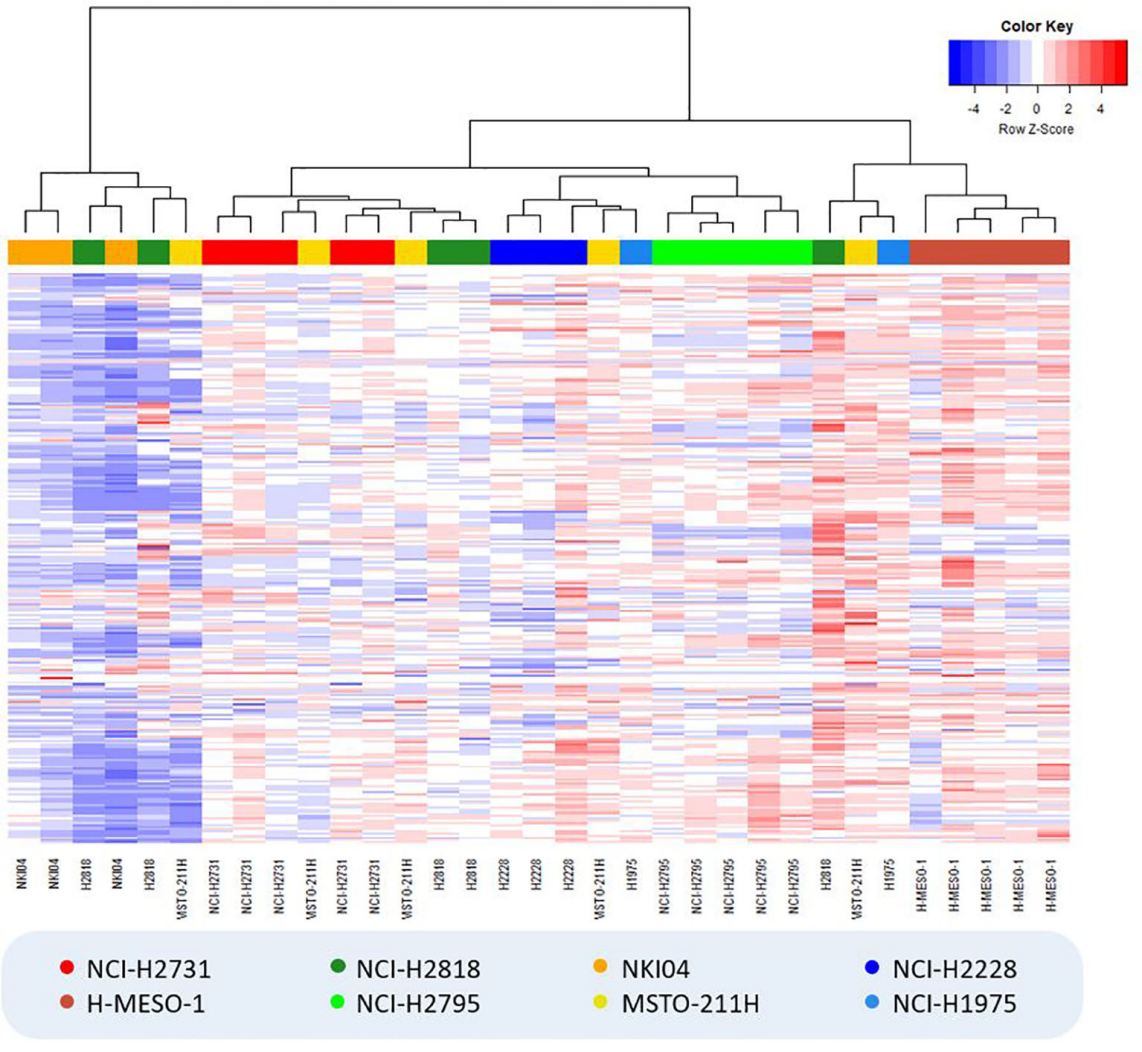
Hierarchically clustered heatmap showing the result of hierarchical clustering analysis (Manhattan distance, Ward’s linkage) for all cell culture samples. VOC relative peak areas are shown in a color gradient, with blue colors indicating less produced VOCs (or more consumed VOCs in the case of negative background-corrected values) compared to the average of all samples (i.e. columns), while red colors indicate the exact opposite. Colors of the cell lines: light/dark green = epithelioid MPM; red/brown = sarcomatoid MPM; yellow/orange = biphasic MPM; dark/light blue = lung cancer.

In the hierarchically clustered heatmap, the VOC profiles tend to naturally cluster together per cell line, demonstrating that each cell line generated a distinct VOC profile (**Figure 2**). Only the cell lines NCI-H2818 and MSTO-211H are more dispersed and show a larger spread around their centroid in the PCA plot, which indicates more variation between the replicates. Remarkably, no clustering could be observed between the two cell lines of the same histological subtype of MPM or lung cancer.

#### 3.2.2 Classification Modeling: Lasso Regression

**Figure 3:**
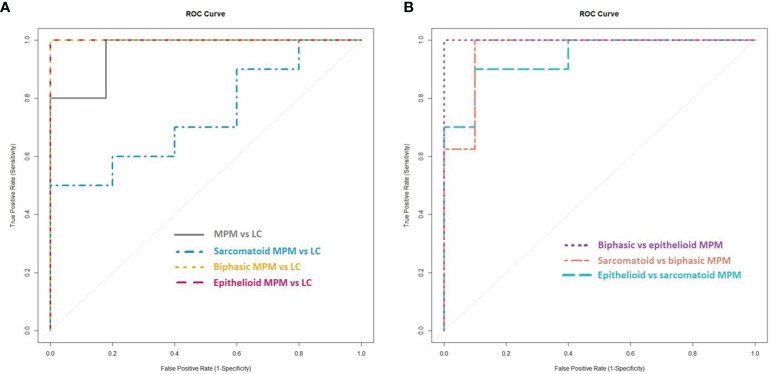
Receiver operating characteristic (ROC) curves of the created lasso models. **(A)** ROC curves of the lasso models for the discrimination between the cell lines of (the histological subtypes of) malignant pleural mesothelioma (MPM) and lung cancer (LC). **(B)** ROC curves of the lasso models for the discrimination between the cell lines of the histological subtypes of MPM.

To identify differentially profiled VOCs between the different cell types, supervised statistical methods can be applied. We used lasso regression to create seven different classification models. The characteristics of the models as well as the selected discriminating VOCs are listed in **Table 2**. The associated ROC curves are shown in **Figure 3**.

**Table 2:** Characteristics of the discrimination models created with least absolute shrinkage and selection operator (lasso) regression and their 95% confidence interval.

	MPM *versus* NSCLC	MPM histological subtype *versus* NSCLC			MPM histological subtype *versus* subtype		
		Epithelioid MPM *versus* NSCLC	Sarcomatoid MPM *versus* NSCLC	Biphasic MPM *versus* NSCLC	Epithelioid *versus* sarcomatoid MPM	Sarcomatoid *versus* biphasic MPM	Biphasic *versus* epithelioid MPM
N	28 *versus* 5	10 *versus* 5	10 *versus* 5	8 *versus* 5	10 *versus* 10	10 *versus* 8	8 *versus* 10
**Sensitivity %**	80.0 (33.5-99.0)	100 (74.1-100)	70.0 (38.0-91.7)	100 (68.8-100)	90.0 (59.7-99.5)	100 (68.8-100)	100 (68.8-100)
**Specificity %**	100 (89.9-100)	100 (54.9-100)	60.0 (18.3-92.6)	100 (54.9-100)	90.0 (59.7-99.5)	90.0(59.7-99.5)	100 (74.1-100)
**Accuracy %**	97.0 (86.0-99.9)	100 (81.9-100)	66.7 (40.1-86.6)	100 (79.4-100)	90.0 (70.8-98.3)	94.4 (75.6-99.7)	100 (84.7-100)
**AUC_ROC_**	0.964 (0.871-1.000)	1.000 (1.000-1.000)	0.740 (0.460-0.960)	1.000 (1.000-1.000)	0.940 (0.820-1.000)	0.962 (0.850-1.000)	1.000 (1.000-1.000)
**VOCs**	propylbenzene, trichloromethane, RT_5.71, RT_9.92, RT_13.77, RT_17.14_C6H12O6, RT_18.94, RT_23.01, RT_24.13, RT_26.28, RT_28.81, RT_30.05, RT_32.39, RT_33.42, RT_33.99, RT_35.44, RT_36.31, RT_36.93, RT_38.35, RT_39.07, RT_39.72, RT_41.03, RT_42.00, RT_46.22_C16H16	methylcyclopentane, n-decane, n-undecane, pentanal, tetradecane, RT_5.34, RT_17.65, RT_21.62, RT_24.01, RT_27.14, RT_29.63, RT_31.45, RT_33.99, RT_36.70, RT_37.92, RT_39.21, RT_41.81	methylcyclopentane, n-decane, n-undecane, pentanal, tetradecane, RT_5.34, RT_17.65, RT_21.62, RT_24.01, RT_27.14, RT_29.63, RT_31.45, RT_33.99, RT_36.70, RT_37.92, RT_39.21, RT_41.81	1-propanol, 1,2,4-trimethylcyclopentane, 1,3-bis(1,1-dimethylethyl)benzene, 2-butanol, 2-methylbutanal, 2-otanone, 3,3-dimethyl-2-butanone, 3-hexanone, 3-undecanone, 5-methyl-3-heptanone, benzaldehyde, cyclohexane, dichloromethane, dodecane, ethylcyclohexane, nonanal, RT_7.73, RT_13.51, RT_17.82, RT_22.30_CH16, RT_23.43, RT_24.13, RT_26.88, RT_28.30, RT_29.15, RT_31.79, RT_33.04, RT_33.35, RT_33.99, RT_34.29, RT_35.57, RT_36.39, RT_36.70, RT_37.76, RT_39.65, RT_39.91, RT_40.77, RT_41.03, RT_42.00, RT_42.49	3-methylpentane, ethylcyclohexane, hexanal, n-undecane, isopropyl nitrate, tetradecane, RT_17.65, RT_29.63, RT_31.84, RT_33.59, RT_37.09, RT_38.71, RT_42.49	2,2,4,4-tetramethyloctane, 5-methyl-3-heptanone, benzene, butanal, dichloromethane, n-undecane, pentanal, propyl nitrate, RT_7.94, RT_18.03, RT_20.70, RT_22.91, RT_24.01, RT_26.28, RT_27.61, RT_29.15, RT_31.45, RT_32.00, RT_33.16, RT_34.36, RT_36.24, RT_36.93, RT_37.66, RT_38.47, RT_39.21, RT_39.91, RT_40.77, RT_41.48, RT_42.53	2,2,4,4-tetramethyloctane, 2,3-dimethylpentane, 2-propanol, 5-methyl-3-heptanone, benzene, butanal, dichloromethane, dodecane, ethyl acetate, n-decane, n-undecane, pentanal, propylbenzene, propyl nitrate, styrene, tetradecane, RT_7.94, RT_17.65, RT_18.03, RT_20.70, RT_22.30_C8H16, RT_22.91, RT_24.01, RT_25.66, RT_26.28, RT_27.61, RT_29.15, RT_31.45, RT_32.00, RT_33.16, RT_34.23, RT_34.36, RT_36.09, RT_36.24, RT_36.93, RT_37.66, RT_38.47, RT_38.93, RT_39.21, RT_39.91, RT_40.77, RT_41.48, RT_42.30, RT_42.53

AUC_ROC_, area under the receiver operating characteristic curve; MPM, malignant pleural mesothelioma; NSCLC, non-small cell lung cancer; RT, retention time; VOC, volatile organic compound.

The shown VOCs were selected in at least 80% of folds.

MPM and lung cancer cells could be clearly differentiated, resulting in 97.0% accuracy, 80.0% sensitivity and 100% specificity. The area under the constructed ROC curve (AUC_ROC_) was 0.964. Twenty-four VOCs were found to be important in this discrimination, of which propylbenzene and trichloromethane could be identified.

New lasso models were constructed for the pairwise comparisons between the three major histological subtypes of MPM and lung cancer. Epithelioid and biphasic MPM cells could be discriminated perfectly from the lung cancer cells with 100% sensitivity, specificity, accuracy and an AUC_ROC_ of 1.000. The sarcomatoid subtype of MPM was less distinguishable from lung cancer, with 70.0% sensitivity, 60.0% specificity, 66.7% accuracy and an AUC_ROC_ of 0.740. The identified VOCs, selected as discriminatory in at least one of the three pairwise comparisons, are; 1-propanol, 1,2,4-trimethylcyclopentane, 1,3-bis(1,1-dimethylethyl)benzene, 2-butanol, 2-methylbutanal, 2-otanone, 3,3-dimethyl-2-butanone, 3-hexanone, 3-undecanone, 5-methyl-3-heptanone, benzaldehyde, cyclohexane, dichloromethane, dodecane, ethylcyclohexane, methylcyclopentane, n-decane, nonanal, n-undecane, pentanal and tetradecane.

To find out whether a differentiation between the different histological subtypes of MPM was also possible, the VOC profiles of the three subtypes were compared to each other. All three subtypes could be differentiated from each other with high sensitivity, specificity and accuracy values (ranging from 90 to 100%) with epithelioid MPM being most distinguishable from biphasic MPM. The identified VOCs that contributed most to at least one of these differentiations are; 2,2,4,4-tetramethyloctane, 3-methylpentane, 2,3-dimethylpentane, 2-propanol, 5-methyl-3-heptanone, benzene, butanal, dichloromethane, dodecane, ethyl acetate, ethylcyclohexane, hexanal, n-decane, n-undecane, pentanal, isopropyl nitrate, propylbenzene, propyl nitrate, styrene and tetradecane.

## 4 Discussion

Clinical studies focusing on VOC biomarkers for MPM have demonstrated the great potential of VOC analysis as a non-invasive, simple and easy-to-use diagnostic tool (11). However, the pathophysiological mechanisms behind alterations in VOC levels are still largely unknown, hampering implementation in clinical practice. In this regard, analysis of MPM cell lines could provide valuable insights into the origin of VOCs and their link to the pathogenesis of MPM, filling in the gaps that still exist today. To our knowledge, we present the first study to report *in vitro* VOC analysis data of all three histological subtypes of MPM. Moreover, this is the first study to identify differential VOCs between MPM and lung cancer cells.

Unsupervised analysis of the TD-GC-MS data showed a clear visual separation between the different cell lines, revealing differences in the VOC profiles generated by the MPM and lung cancer cells (**Figure 1**). Furthermore, the hierarchically clustered heatmap did not show any cell culture media-based clustering, indicating the effectiveness of the applied background correction (**Figure 2**). Surprisingly, neither both cell lines of the same histological subtype of MPM nor both lung cancer cell lines did seem to cluster very closely together, implying that each individual cell line generates a unique VOC profile. These observations are in line with the findings of Peled et al. (23), who observed differences in VOC profile between individual lung cancer cell lines from the same histological subtype, but with different genetic mutations, including both lung adenocarcinoma cell lines used in our study. It is therefore possible that our observed differences are also caused by genetic variation.

Many of the discriminative VOCs that are identified in studies concerning a specific type of cancer are also described in studies involving other cancer types (24–26). Therefore, comparison between different types of cancer is of utmost importance to pinpoint VOC profiles that are specific for the tumor of interest and are not just related to cancer in general (24). This was addressed in our study by discriminating MPM and lung cancer cells with 97% accuracy (**Table 2**). Only Gendron et al. (18) previously described the distinction between an MPM cell line and lung cancer cell lines, using eNose. However, no individual VOCs were identified and no performance characteristics were reported in their study, allowing no direct comparison. With 97% accuracy, our *in vitro* model even outperformed the *in vivo* situation where MPM patients could be discriminated from lung cancer patients with only 72% accuracy (13). Such an accurate distinction between two types of thoracic malignancies emphasizes the difference in metabolic profile which is important for the clinical utility of VOCs as biomarkers for differential diagnosis. Twenty-four VOCs were found to be important in this *in vitro* discrimination, of which only propylbenzene and trichloromethane could be identified. Propylbenzene has been previously described as a possible biomarker for lung cancer both *in vitro* and *in vivo* (27, 28). This is the first time a significant difference in propylbenzene abundance between MPM and lung cancer cells is reported, demonstrating the potential of this compound as discriminator. The second compound, trichloromethane, has already been identified as possible breath biomarker to discriminate MPM patients from asbestos-exposed individuals in a clinical study (14). This *in vitro* observation confirms a possible relationship between this compound and MPM. However, since trichloromethane could also be a solvent contamination, its interpretation should be done carefully and requires further investigation.

Regarding the subtypes of MPM, both epithelioid and biphasic MPM cells could be discriminated from the lung cancer cells with 100% accuracy (**Table 2**). The distinction between the sarcomatoid subtype and lung cancer appeared to be less clear with an accuracy value of 66.7%, suggesting that these cell types have a more similar volatile fingerprint which may result from the activity of similar pathways. However, as the epithelioid and biphasic subtype account for approximately 90% of all MPM cases, a correct distinction between lung cancer and the two most prevalent subtypes is a promising outcome (5).

Alkanes and aldehydes represent many of the discriminatory VOCs when comparing MPM histological subtypes with lung cancer. These compounds can result from cell membrane phospholipid peroxidation, caused by the large amount of radicals produced in the tumor cells (oxidative stress) (29). It has been stated that the phospholipid composition of lung cancer cells is different compared to non-malignant cells, implying that lipid peroxidation may cause production of cancer-specific VOC profiles (30). Some of the selected compounds like n-undecane, pentanal, n-decane and methylcyclopentane have already been identified in other studies as lung cancer biomarkers, confirming their possible discriminatory properties (28, 31). In one of our previous studies, methylcyclopentane was even selected as potential biomarker for MPM when comparing exhaled breath from patients with that of asbestos-exposed persons, suggesting that the concentration of this compound might differentiate between at-risk controls, MPM patients and lung cancer patients (14). Additionally, different ketones are among the selected discriminators, which is not unexpected as they are known to be related to the increased oxidation rate of fatty acids during carcinogenesis. Furthermore, in many cancer types a significantly higher activity of alcohol dehydrogenase is observed, which oxidizes alcohols to ketones (24).

Differentiation between the three MPM subtypes could also be achieved with high accuracy (ranging from 90 to 100%), (**Table 2**). Since the epithelioid and sarcomatoid subtype are associated with the best and worst prognosis respectively, determining VOCs in exhaled breath could potentially have a prognostic value (5). To date, no clinical studies have been carried out comparing the breath VOC profile of MPM patients with different histological subtypes, since MPM is a rare disease and the epithelioid subtype is the most prevalent. Little et al. (19) are the only other group, besides Gendron et al., that analyzed the headspace of MPM cells. They compared one biphasic MPM (MSTO-211H), one epithelioid MPM (NCI-H28) and one non-malignant mesothelial (MET-5A) cell line using solid-phase microextraction GC-MS (19). They identified 2-ethyl-1-hexanol to be significantly increased in both MPM cell lines compared to the non-malignant cell line. In addition, ethyl propionate and cyclohexanol were seen to be specifically increased in the biphasic MPM cell line, while dodecane was only increased in the epithelioid MPM cell line. In line with these findings, dodecane was also selected in our study as an important discriminator between biphasic and epithelioid MPM, suggesting a potential role as subtype-specific marker. However, dodecane was also found to be related to lung cancer and breast cancer in other studies, implying it could also be a more general cancer marker (27).

Our study has several strengths compared to the previous studies analyzing the headspace of MPM cells. Firstly, to cover the natural heterogeneity of the disease, we included six different MPM cell lines of different histological subtypes, rather than replicates of only one or two cell lines. Differences in number of cells were to be expected due to differences in cell size and growth rate, which is why we opted for obtaining equal metabolic surface areas after the incubation period, rather than an equal number of cells. Secondly, unlike many other VOC studies, we have chosen not to rule out unidentified compounds since these could also be important discriminators. This is demonstrated by the considerable number of unidentified VOCs selected by the regression models. This number indicates that there is still room for improvement of analytical techniques to achieve maximum VOC identification, which should certainly be addressed in future research. Lastly, given the high number of VOCs that are present in human matrices and the fact that numerous VOCs seem to be of importance in different diseases, a VOC panel rather than a single biomarker should be used for MPM diagnosis (11). By performing multivariate statistics, the optimal combination of VOCs (VOC patterns) is selected to distinguish the indicated groups. This is a more suitable approach than applying univariate statistics, as applied by the group of Little (19), which focusses on individual VOCs that may lack specificity when it comes to other diseases (32). It is important to further validate these identified VOC profiles by involving a wider range of diseases.

Several clinical studies proposed different VOCs as breath biomarkers for MPM, however, only limited overlap is seen with our *in vitro* results (13–15, 33, 34). These discrepancies between *in vitro* and *in vivo* findings can be the result of changes in cell metabolism due to differences in oxygen levels or standard 2D culturing conditions (35, 36). Furthermore, the transmission of VOCs from cells to breath is poorly understood, involving possible conversion of compounds by the liver or kidney metabolism (36, 37). These factors make *in vitro* and *in vivo* results difficult to compare, stressing the need for studies investigating simultaneously breath and tumor cells of the same patients and investigating metabolization and kinetics of *in vitro* discovered VOCs in the *in vivo* setting.

Despite the added value of our study, these discrepancies lead us to some study limitations that should be recognized. The experiments were performed under standard 2D culturing conditions, disregarding the 3D structure and oxygen deficient tumor microenvironment *in vivo.* More advanced, specialized set-ups, better mimicking *in vivo* conditions, should be used in further studies when available. Secondly, we have chosen not to make a comparison between malignant and non-malignant mesothelial cells, since our focus was on the specificity of the VOC biomarkers. Therefore, we used lung cancer cells as control group. Little et al. (19) alternatively used normal mesothelial cells in their experimental set-up. However, the sparsely commercially available normal mesothelial cell lines are not considered well representative of *in vivo* mesothelial cells (38). Hence, such cells might not be an accurate normal cell control for MPM. As an alternative, primary mesothelial cells could be isolated from human pleura, but they have the disadvantage of being difficult to cultivate *in vitro*, limiting their use for *in vitro* headspace analysis. A final potential limitation relates to the possible exogenous origin of VOCs. Although a background correction was made to correct for exogenous VOCs originating from the used culture media, materials and sampling environment, this does not guarantee that all measured VOCs are of endogenous origin. This should always be kept in mind when interpreting the results. However, despite these possible limitations, the presented study shows new and valuable results as one of the first studies to investigate VOC production at the cellular level for MPM.

## 5 Conclusions

Breath analysis has proven to be a promising tool for the non-invasive diagnosis of MPM. To gain insight into the biological processes underlying VOC production, *in vitro* VOC analysis can provide valuable additional information. This study identified MPM-specific VOC profiles capable of differentiating MPM subtypes and lung cancer cells with high accuracy. However, discrepancies between these identified *in vitro* VOC profiles and clinically reported breath profiles were observed, which could be explained by differences in oxygen levels, 3D structure, metabolization, etc. This supports the need for further investigation of these *in vitro* discovered VOCs and their metabolic pathways as well as their kinetics *in vivo*. While the relationship between *in vitro* and *in vivo* VOCs is still largely unknown, both could complement each other in generating a clinically useful breath model for MPM.

## Data Availability Statement

The raw data supporting the conclusions of this article will be made available by the authors, without undue reservation.

## Author Contributions

Conceptualization, KV, JvM, and KL; data curation, EJ and ZM; formal analysis, EJ and ZM; investigation, EJ, ZM, LV, and SL; methodology, EJ, ZM, LV, SL, KV, CW, EM, and KL; supervision, JvM, EM, and KL; visualization, EJ; writing – original draft preparation, EJ; writing – review and editing, EJ, ZM, LV, KV, JvM, CW, EM, and KL. All authors have read and agreed to the published version of the manuscript.

## Funding

This research was funded by Kom op tegen Kanker (Stand up to Cancer), the Flemish cancer society (grants KOTK UA/2016/10710/1 and ‘Emmanuel van der Schueren’) and by the Antwerp University Research Fund (BOF-KP 36051).

## Conflict of Interest

The authors declare that the research was conducted in the absence of any commercial or financial relationships that could be construed as a potential conflict of interest.

## Publisher’s Note

All claims expressed in this article are solely those of the authors and do not necessarily represent those of their affiliated organizations, or those of the publisher, the editors and the reviewers. Any product that may be evaluated in this article, or claim that may be made by its manufacturer, is not guaranteed or endorsed by the publisher.
