# Supplementary figures and images for: Headspace Volatile Organic Compound Profiling of Pleural Mesothelioma and Lung Cancer Cell Lines as Translational Bridge for Breath Research
Source: Front Oncol. 2022 May 6;12:851785. doi: 10.3389/fonc.2022.851785 (PMC9120820; doi:10.3389/fonc.2022.851785)

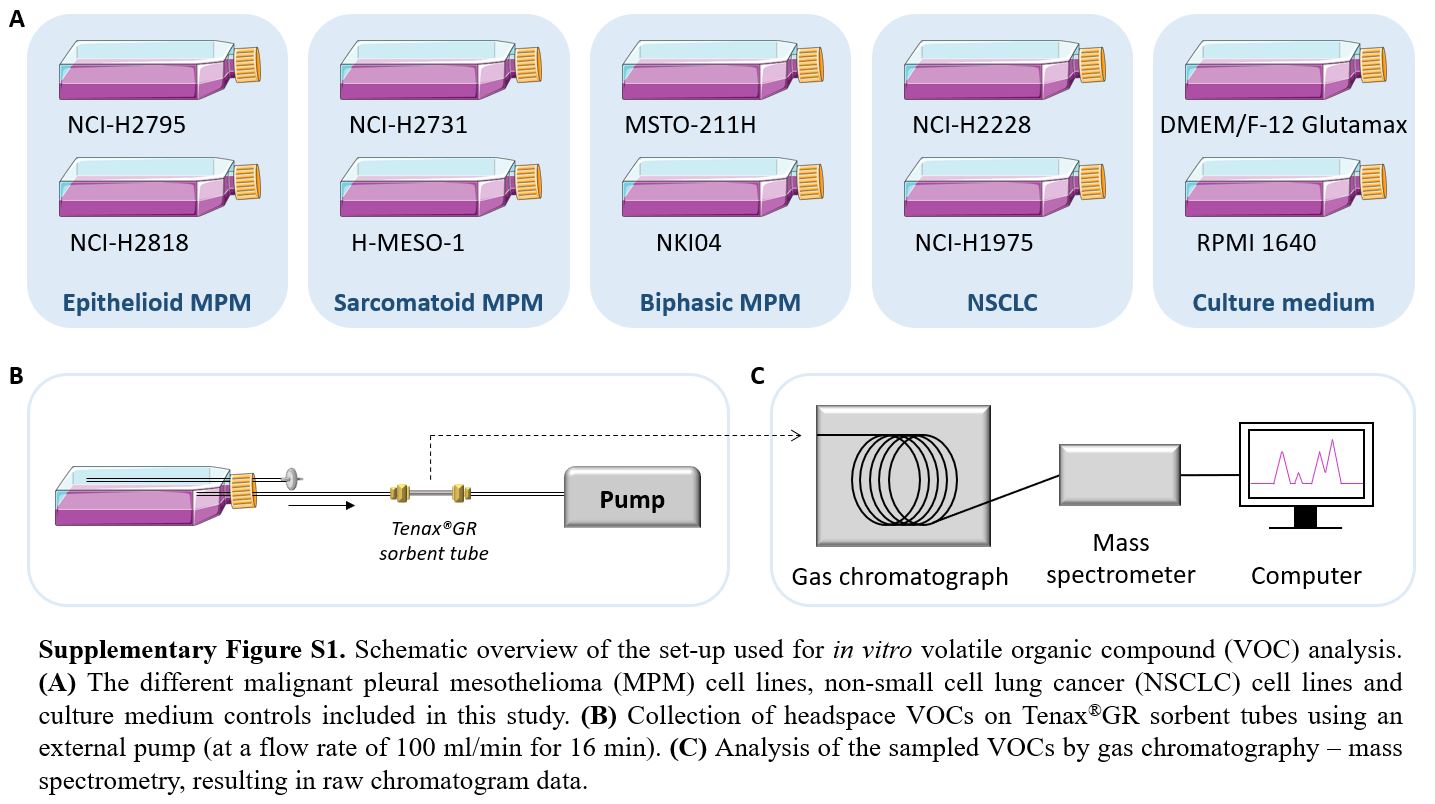

Supplement: Supplementary file 1 [file Image_1.jpeg]
