# Supplementary material for: Headspace Volatile Organic Compound Profiling of Pleural Mesothelioma and Lung Cancer Cell Lines as Translational Bridge for Breath Research
Source: Front Oncol. 2022 May 6;12:851785. doi: 10.3389/fonc.2022.851785 (PMC9120820; doi:10.3389/fonc.2022.851785)
Supplement: Supplementary file 2 [file Table_1.docx]

**Supplementary Table S1.** Overview of the 77 identified volatile organic compounds (VOCs) categorized into eleven chemical classes.

| Chemical class | VOCs |
| --- | --- |
| Alcohols | ethanol, 2-propanol, 2-methyl-2-propanol, 2-methyl-2-propanal, 1‑propanol, 2-butanol, 1-butanol, 2-ethyl-1-hexanol, 2-methyl-2-butanol |
| Aldehydes | acetaldehyde, butanal, 3-methylbutanal, 2-methylbutanal, pentanal, hexanal, benzaldehyde, octanal, nonanal |
| Aliphatic hydrocarbons | butane, pentane, 2-methylpentane, 3-methylpentane, 1-hexene, hexane, methylcyclopentane, 3,3-dimethylpentane, cyclohexane, 2-methylhexane, 2,3-dimethylpentane, 3-methylhexane, heptane, methylcyclohexane, 1,2,4-trimethylcyclopentane, 2-methylheptane, 3-methylheptane, octane, ethylcyclohexane, nonane, n-decane, 2,2,4,4-tetramethyloctane, n‑undecane, dodecane, tridecane, tetradecane |
| Aromatic hydrocarbons | benzene, toluene, ethylbenzene, meta-/para-xylene, styrene, propylbenzene, 1,3-bis(1,1-dimethylethyl)benzene |
| Esters | ethyl acetate |
| Halogenated compounds | dichloromethane, trichloromethane, 1H,1H,2H,2H-perfluoro-1-octanol |
| Ketones | acetone, 2-butanone, 3-methyl-2-butanone, 2-pentanone, 3-pentanone, 3,3-dimethyl-2-butanone, 3-methyl-2-pentanone, 3-hexanone, 2-hexanone, 3-methyl-4-heptanone, 5-methyl-3-heptanone, 2-octanone, acetophenone, 3-decanone, 3-undecanone |
| Nitrogen compounds | nitromethane, isopropyl nitrate, propyl nitrate |
| Siloxanes | hexamethylcyclotrisiloxane, octamethylcyclotetrasiloxane |
| Sulphides | carbondisulphide |
| Terpenes | limonene |
